# Phenotypic differences between female and male individuals with suspicion of autism spectrum disorder

**DOI:** 10.1186/s13229-022-00491-9

**Published:** 2022-03-07

**Authors:** Sanna Stroth, Johannes Tauscher, Nicole Wolff, Charlotte Küpper, Luise Poustka, Stefan Roepke, Veit Roessner, Dominik Heider, Inge Kamp-Becker

**Affiliations:** 1 Department of Child and Adolescent Psychiatry, Psychosomatics and Psychotherapy, Faculty of Human Medicine, Philipps University Marburg, Hans-Sachs Str. 6, 36037 Marburg, Germany; 2 Department of Mathematics and Computer Science, Philipps University Marburg, Hans-Meerwein-Straße 6, 35043 Marburg, Germany; 3 Department of Child and Adolescent Psychiatry, TU University Dresden, Fetscherstr. 74, 01307 Dresden, Germany; 4 Department of Psychiatry, Charité - Universitätsmedizin Berlin, Campus Benjamin Franklin, Hindenburgdamm 30, 12203 Berlin, Germany; 5 Department of Child and Adolescent Psychiatry, University Medical CenterGöttingen, Von-Siebold-Str. 5, 37075 Göttingen, Germany

**Keywords:** Female autism, Sex, ASD, ADOS, ADI-R, Diagnostics, Phenotype

## Abstract

**Background:**

Although autism spectrum disorder (ASD) is a common developmental disorder, our knowledge about a behavioral and neurobiological female phenotype is still scarce. As the conceptualization and understanding of ASD are mainly based on the investigation of male individuals, females with ASD may not be adequately identified by routine clinical diagnostics. The present machine learning approach aimed to identify diagnostic information from the Autism Diagnostic Observation Schedule (ADOS) that discriminates best between ASD and non-ASD in females and males.

**Methods:**

Random forests (RF) were used to discover patterns of symptoms in diagnostic data from the ADOS (modules 3 and 4) in 1057 participants with ASD (18.1% female) and 1230 participants with non-ASD (17.9% % female). Predictive performances of reduced feature models were explored and compared between females and males without intellectual disabilities.

**Results:**

Reduced feature models relied on considerably fewer features from the ADOS in females compared to males, while still yielding similar classification performance (e.g., sensitivity, specificity).

**Limitations:**

As in previous studies, the current sample of females with ASD is smaller than the male sample and thus, females may still be underrepresented, limiting the statistical power to detect small to moderate effects.

**Conclusion:**

Our results do not suggest the need for new or altered diagnostic algorithms for females with ASD. Although we identified some phenotypic differences between females and males, the existing diagnostic tools seem to sufficiently capture the core autistic features in both groups.

**Supplementary Information:**

The online version contains supplementary material available at 10.1186/s13229-022-00491-9.

## Background

Autism spectrum disorder (ASD) is a common developmental disorder with an onset within the first years of life and early emerging atypicalities in social attention and reciprocity [1]. Since the early days of autism research, the condition has been understood to predominantly affect males. Most epidemiological studies report an approximately 4:1 male to female ratio [2, 3], which has recently shifted toward a 3:1 ratio [4]. It has consistently been shown that the sex imbalance in prevalence varies with cognitive ability, with a male to female ratio of 2:1 among individuals with low cognitive ability or co-occurring intellectual disability and a ratio as high as 9:1 among individuals with average to above-average IQ [2, 5]. Consequently, most research has involved males, leading to a male-biased understanding and conceptualization of ASD. Furthermore, although the female ASD phenotype may present differently, current defining diagnostic criteria are mainly based on male characteristics, and diagnostic instruments may be biased toward detecting ASD among male individuals, with similar diagnostic thresholds for females and males [6, 7].

Previous work suggests that ASD may be more difficult to detect in females, as they tend to be diagnosed later than males [8, 9] and seem to require a more significant etiological load to manifest autistic behavioral characteristics and autistic symptoms, or concurrent impairments need to be more severe for the diagnosis to be given [10]. It is argued that phenotypic sex differences might lead to delayed or even missed diagnoses in girls and women with ASD [11].

In the past decades, a wealth of investigations have been conducted to examine the relationship between sex and clinical profiles of individuals with ASD. Research findings on differences between the sexes provide some insight into why females might be more difficult to detect and are diagnosed later in life than males. However, knowledge about differences between the sexes in terms of the phenotypic presentation of ASD symptoms is still lacking, as the available studies yielded inconsistent findings regarding symptom severity across different age groups and different levels of functioning. While some studies did not find sex differences during a behavioral observation, e.g., [12, 13], others did report some differences [14].

It has been argued that females and males might meet the diagnostic criteria for ASD differently, as a range of different behaviors can be mapped onto each broad criterion. For example, deficits in social-emotional reciprocity may be composed of impairments in spoken language, reduced joint attention, and reduced sharing of interest, emotions, and affect. To meet the social-emotional reciprocity criterion, an individual does not need to present with all of these behaviors—rather, the clinician needs to decide whether or not an individual meets a particular criterion based on the available information. Despite some work on a female autism phenotype, little is known about how females and males meet the diagnostic criteria.

Females with ASD appear to score lower than males on measures of restricted and repetitive behavior (RRB), they seem less likely to present with stereotyped use of objects and show different types of restricted interests than males [15]. Specific differences in social communication deficits have not been consistently observed. Some girls were more likely to show an ability to integrate non-verbal and verbal behaviors, maintain a reciprocal conversation, and be able to initiate, but not maintain friendships [15] others showed more impairment in communication [16] compared to boys. Overall, results remain inconsistent (for reviews, see [5, 17–19]. If there are indeed different symptom patterns in females and males, but diagnostic instruments are biased toward the male ASD phenotype, one solution to better recognize ASD in females would be to revise the diagnostic criteria and the diagnostic algorithms of standard diagnostic instruments.

The current study aimed to investigate an ASD specific behavioral observation tool and explore whether there are differences in how female and male individuals meet the actual criteria for ASD. As females with ASD without cognitive and language deficits are at risk of not being identified until later in life, we investigated a sample of individuals with fluent language and without profound intellectual disabilities. Diagnoses at an older age have been associated with increased comorbidity [20]. Moreover, the presentation of ASD symptoms can significantly overlap with other mental disorders [21, 22]. Therefore, it is essential to investigate sex differences in ASD symptom presentation not only in a sample of individuals with ASD but also in those with suspicion of ASD but no actual ASD diagnosis. The present study thus aimed to extend previous research on a female ASD phenotype, which focused on differences in ASD symptoms between females and males already diagnosed with ASD, by including a large clinical sample comprising individuals without a diagnosis of ASD but with a diagnosis of other mental disorders. We aimed to identify those symptoms, directly observed by trained specialists, which optimally discriminate between ASD and non-ASD within a female and a male sample, and to then compare these discriminative features between the sexes. We thus aim to facilitate the diagnostic identification of females by highlighting potential nuanced differences between the sexes. By using machine learning models, we sought to identify the particular contributions of individual pieces of diagnostic information (item codes from the ADOS) for the diagnosis of female and male children and young adolescents and for later diagnosis of older adolescents and adults.

## Methods

### Participants

Datasets were drawn from medical records (retrospective chart review of the period between 2000 and 2019) from five specialized autism centers that were part of the ASD-Net, a research consortium funded by the German Federal Ministry of Education and Research (BMBF) [22] and coordinated by the authors. Experienced clinicians with continuous ADOS coding experience and research reliable ADOS experts for supervision at each site applied the current diagnostic gold standard.

All data were analyzed anonymously, with approval from the local ethics committee (Az. 92/20). Due to the retrospective nature of data collection and analysis based on anonymized data, the need for informed consent was waived by the ethics committee. All methods were applied following relevant institutional and international research guidelines and regulations. Sex is defined as biological sex as assessed by caregivers or the participants themselves. If sex was unknown or not reported, data were excluded (*N* = 4).

The dataset included 2287 individuals who underwent a complete clinical examination after an initial suspicion of ASD. 46.2% received a diagnosis of ASD (*n* = 866 male; *n* = 191 female). The remaining participants (53.8%) did not receive a diagnosis of ASD, and were diagnosed with other mental or developmental disorders or no disorders (*n* = 1010 male; *n* = 220 female). The non-ASD group represents a well-balanced data set comprising differential disorders with some traits or symptoms of ASD (leading to the suspicion of ASD). The ratio of males to females with ASD was 4.5:1. The sample was separated into subsamples, as participants were administered different measures (ADOS modules) depending on age and language ability. The subsamples are henceforth labeled “*children and young adolescents*” (examined with ADOS module 3) and “*older adolescents and adults*” (examined with ADOS module 4).

#### Child and young adolescent sample

Of the children and young adolescents with ASD, 51% had further co-occurring diagnoses (most commonly attention deficit hyperactivity disorder (ADHD, F90 according to ICD-10) and developmental disorders (F80 and F82). The most common non-ASD diagnoses were ADHD (23%) and conduct disorders (11%). 79% had further co-occurring diagnoses. In contrast to this high multi-comorbidity, 22% of non-ASD cases had no clinical mental disorder but did have some autistic traits that had led to the suspicion of ASD. Further details can be found in Additional file 1: Tables S1 and S2.

On average, the non-ASD group was younger than the ASD group (*T*(152) = 2.24, *p* = 0.027 for females, *T*(1196) = 2.77, *p* = 0.006) for males), with small to moderate effect sizes (Cohen’s *d* = 0.38 and 0.16) (see Table 1). Comparing only those female and male individuals with an ASD diagnosis (see Table 2), females were older than males (*T*(545) = 2.8, *p* = 0.005; *mean* = 11.4, *median* = 11, *SD* = 3.2 in females and *mean* = 10.3, *median* = 10, *SD* = 2.8 in males), with a moderate effect size (*d* = 0.40). There were no differences concerning IQ.

**Table 1:** Sample characteristics

	ASD		Non-ASD		*t* test			ES
	*N*	*M* (SD)	*N*	*M* (SD)	*t*	*df*	*p*	
*Female children and young adolescents*								
Age	52	11.46 (3.23)	102	10.30 (2.94)	2.24	152	**.027**	**0.38**
IQ	47	93.81 (17.88)	66	97.91 (19.54)	1.14	111	.257	0.22
ADOS SA	52	8.79 (4.23)	102	2.65 (3.27)	9.96	152	**.000**	**1.62**
ADOS RRB	52	1.19 (1.36)	102	0.31 (0.69)	5.33	152	**.000**	**0.82**
ADI-R A	33	14.58 (6.15)	27	7.89 (4.77)	4.63	58	**.000**	**1.22**
ADI-R B	33	10.30 (4.86)	27	6.37 (4.86)	3.30	58	**.002**	**0.81**
ADI-R C	33	3.30 (1.85)	27	2.00 (1.36)	3.05	58	**.003**	**0.80**
*Male children and young adolescents*								
Age	495	10.29 (2.79)	703	9.86 (2.58)	2.77	1196	**.006**	**0.16**
IQ	408	97.68 (18.15)	521	98.50 (18.35)	0.68	927	.498	0.05
ADOS SA	495	9.95 (4.09)	703	3.23 (3.53)	30.30	1196	**.000**	**1.76**
ADOS RRB	495	1.48 (1.37)	703	0.31 (0.59)	20.14	1196	**.000**	**1.11**
ADI-R A	339	16.40 (5.98)	253	9.53 (5.91)	13.89	590	**.000**	**1.16**
ADI-R B	339	12.69 (5.17)	253	6.67 (4.32)	15.03	590	**.000**	**1.26**
ADI-R C	339	4.27 (2.43)	253	2.08 (1.69)	12.35	590	**.000**	**0.65**
Age	495	10.29 (2.79)	703	9.86 (2.58)	2.77	1196	**.006**	**0.16**
*Female older adolescents and adults*								
Age	139	29.80 (11.56)	118	29.79 (12.64)	0.006	254	.995	0.00
IQ	128	106.78 (14.34)	98	105.05 (14.12)	0.905	224	.366	0.12
ADOS SA	139	9.35 (4.22)	118	2.96 (3.13)	13.58	255	**.000**	**1.70**
ADOS RRB	139	1.33 (1.25)	118	0.28 (0.63)	8.24	255	**.000**	**1.06**
ADI-R A	72	13.18 (7.01)	42	5.60 (4.64)	6.22	112	**.000**	**1.27**
ADI-R B	72	8.29 (4.97)	42	3.14 (3.75)	5.82	112	**.000**	**1.17**
ADI-R C	72	2.97 (2.44)	42	1.02 (1.30)	4.78	112	**.000**	**1.00**
*Male older adolescents and adults*								
Age	371	24.87 (10.61)	307	26.46 (12.02)	1.82	676	.069	0.14
IQ	326	103.36 (17.00)	250	104.18 (15.56)	0.59	574	.553	0.05
ADOS SA	371	10.11 (4.21)	307	4.37 (3.88)	18.33	676	**.000**	**1.42**
ADOS RRB	371	1.63 (1.37)	307	0.69 (0.90)	10.30	676	**.000**	**0.81**
ADI-R A	205	13.98 (6.05)	136	6.59 (5.26)	11.62	339	**.000**	**1.30**
ADI-R B	205	10.00 (4.88)	136	4.21 (3.47)	11.97	339	**.000**	**1.37**
ADI-R C	205	3.09 (2.10)	136	1.39 (1.40)	8.29	339	**.039**	**0.95**

Bold font indicates statistical significance on a .05 level

*IQ* intelligence quotient, *ADOS*  Autism Diagnostic Observation Schedule, *SA* social affect, *RRB* restricted repetitive behaviors, *ADI*  autism diagnostic interview, *ADI-R A*  social interaction, *ADI-R B*  communication, *ADI-R C * restricted repetitive behaviors, *ES* effect size (Cohen’s *d*)

**Table 2:** Characteristics of the *ASD sample* for males and females separately

	*N*	Female	*N*	Male	*T*	*df*	*p*	ES
*Children and young adolescents*								
Age	52	11.5 (3.2)	495	10.3 (2.8)	2.8	545	**.005**	**0.40**
IQ	47	93.8(17.9)	408	97.7 (18.2)	1.4	453	.167	0.22
ADOS-SA	52	8.8 (4.2)	495	9.9 (4.1)	1.9	545	.054	0.27
ADOS-RRB	52	1.2 (1.4)	495	1.5 (1.4)	1.4	545	.155	0.21
ADOS-CSS	52	5.8 (2.5)	495	6.6 (2.4)	2.4	545	**.016**	**0.33**
ADI_A	33	14.6 (6.2)	339	16.4 (6.0)	17	370	.096	0.30
ADI_B	33	10.3 (4.9)	339	12.7 (5.2)	2.5	370	**.011**	**0.48**
ADI_C	33	3.3 (1.9)	339	4.3 (2.4)	2.2	370	**.026**	**0.46**
*Older adolescents and adults*								
Age	139	29.8 (11.5)	371	24.9 (10.6)	4.5	507	**.000**	**0.44**
IQ	128	106.8 (14.4)	326	103.6 (17)	2.0	452	**.045**	**0.20**
ADOS-SA	139	9.3 (4.2)	371	10.11 (4.2)	1.8	508	.068	0.19
ADOS-RRB	139	1.3 (1.2)	371	1.6 (1.4)	2.3	508	**.023**	**0.23**
ADOS-CSS	139	5.7 (2.6)	371	6.2 (2.5)	2.3	508	**.024**	**0.20**
ADI_A	72	13.2 (7.0)	205	14.0 (6.1)	0.6	234	.550	0.12
ADI_B	72	8.3 (5.0)	205	10.0 (4.9)	2.4	233	**.016**	**0.34**
ADI_C	72	3.0 (2.4)	205	3.1 (2.1)	1.8	40	.076	0.04

Bold font indicates statistical significance on a .05 level

*IQ* intelligence quotient, *ADOS*  Autism Diagnostic Observation Schedule, *ADOS-SA* social affect, *ADOS-RRB*  restricted repetitive behaviors, *ADOS-CSS*  comparison score, *ADI*  autism diagnostic interview, *ADI-R A * social interaction, *ADI-R B*  communication, *ADI-R C*  restricted repetitive behaviors, *ES* effect size (Cohen’s *d*)

#### Older adolescent and adult sample

In older adolescents and adults with ASD, 51% had co-occurring diagnoses (most commonly depressive disorders, in 25% of the sample). The most common non-ASD diagnoses were affective disorders (21%) and personality disorders (20.5%). 72% had further co-occurring diagnoses. Again, in contrast to this high multi-comorbidity, 35% of non-ASD cases had no clinical mental disorder. Further details can be found in Additional file 1: Tables S1 and S2.

There were no differences between the ASD and non-ASD groups with regard to age or IQ (see Table 1). Comparing only those female and male individuals with an ASD diagnosis (see Table 2), again, females were older than males on average (*T*(507) = 4.58, *p* = 0.000; *mean* = 29.8, *median* = 28, SD = 11.5 in females and *mean* = 24.9, *median* = 22, *SD* = 10.6 in males, *d* = 0.44), and females had a slightly higher full IQ (*T*(452) = 2.0, *p* = 0.045, *d* = 0.20).

### Measures

The Autism Diagnostic Observation Schedule (ADOS-G/ADOS-2) [23, 24] is a standardized instrument that assesses social interaction, communication, and imagination during a semi-structured interaction with an examiner. It is an internationally used diagnostic instrument that consists of a module for toddlers and four additional modules to be administered based on the individual’s level of expressive language, chronological age, and appropriateness of the respective assessment materials. ADOS codes indicate symptom severity by coding increasing severity with codes of 0, 1, 2, and 3. Specific ADOS codes additionally contain information about peculiar or abnormal behavior using codes 7 or 8. There are 29 behavioral aspects (very specific aspects such as eye contact and broader aspects such as quality of social overtures) that have to be observed and coded in Module 3, and 31 behavioral codes in Module 4, of which 14 are entered into the respective classification algorithm. The ADOS provides diagnostic cut-offs for “no autism,” “autism spectrum,” and “autism” and metrics of ASD symptom severity (“comparison score,” CSS) [23].

For the best estimate clinical diagnosis (BEC), the ADOS needs to be complemented by the Autism Diagnostic Interview—Revised (ADI-R [25], a structured clinical caregiver interview that mostly focuses on ASD-related symptoms at the age of 4.0–5.0 years. The scoring of the ADI-R is organized into three behavioral domains: qualitative abnormalities in reciprocal social interaction (A); qualitative abnormalities in communication (B); and restricted and repetitive behavior (C). Furthermore, a careful differential diagnostic examination, physical examination, medical history-taking, and assessment of intellectual abilities are required for BEC [26] and were undertaken in the present study using standard diagnostic instruments. All diagnoses in the current study were built on a thorough clinical characterization of all individuals, leading to BEC diagnoses that did not always correspond to the classification according to the ADOS diagnostic cut-offs (see fourfold Table 3 for details).

**Table 3:** Fourfold table of ASD/non-ASD versus ADOS cut-offs

		Non-autism	Spectrum	Autism
*Children and young adolescents*				
Female				
BEC				
Non-ASD	N	86 (84.3%)	5 (4.9%)	11 (10.8%)
ASD	N	10 (19.2%)	12 (23.1%)	30 (57.7%)
Male				
BEC				
Non-ASD	N	571 (81.2%)	64 (9.1%)	68 (9.7%)
ASD	N	61 (12.3%)	74 (14.9%)	360 (72.7%)
*Older adolescents and adults*				
Female				
BEC				
Non-ASD	N	103 (87.3%)	6 (5.1%)	9 (7.6%)
ASD	N	25 (18.0%)	24 (17.3%)	90 (67.7%)
Male				
BEC				
Non-ASD	N	220 (71.7%)	34 (11.1%)	53 (17.3%)
ASD	N	55 (14.8%)	43 (11.6%)	273 (73.6%)

*BEC* best estimate clinical diagnoses, *ASD*  autism spectrum disorder

### Data analysis

To explore differences in demographic characteristics between females and males, t-tests were conducted. To explore which ADOS items discriminate best between ASD and non-ASD within the two groups of females and males, we trained a random forest (RF) algorithm. Twenty-eight items of the ADOS module 3 and 31 items of the ADOS module 4 entered as predictors (item Amount of Social Overtures/ Maintenance of Attention to Examiner of ADOS-2 was excluded because it is only used in ADOS-2 and was not available for cases examined with ADOS-G). Following the ADOS manual instructions, we remapped codes of 7 and 8 to 0, and codes of 3 were recoded to 2. The ASD best estimate clinical diagnosis was the classification criterion.

**Fig. 1:**
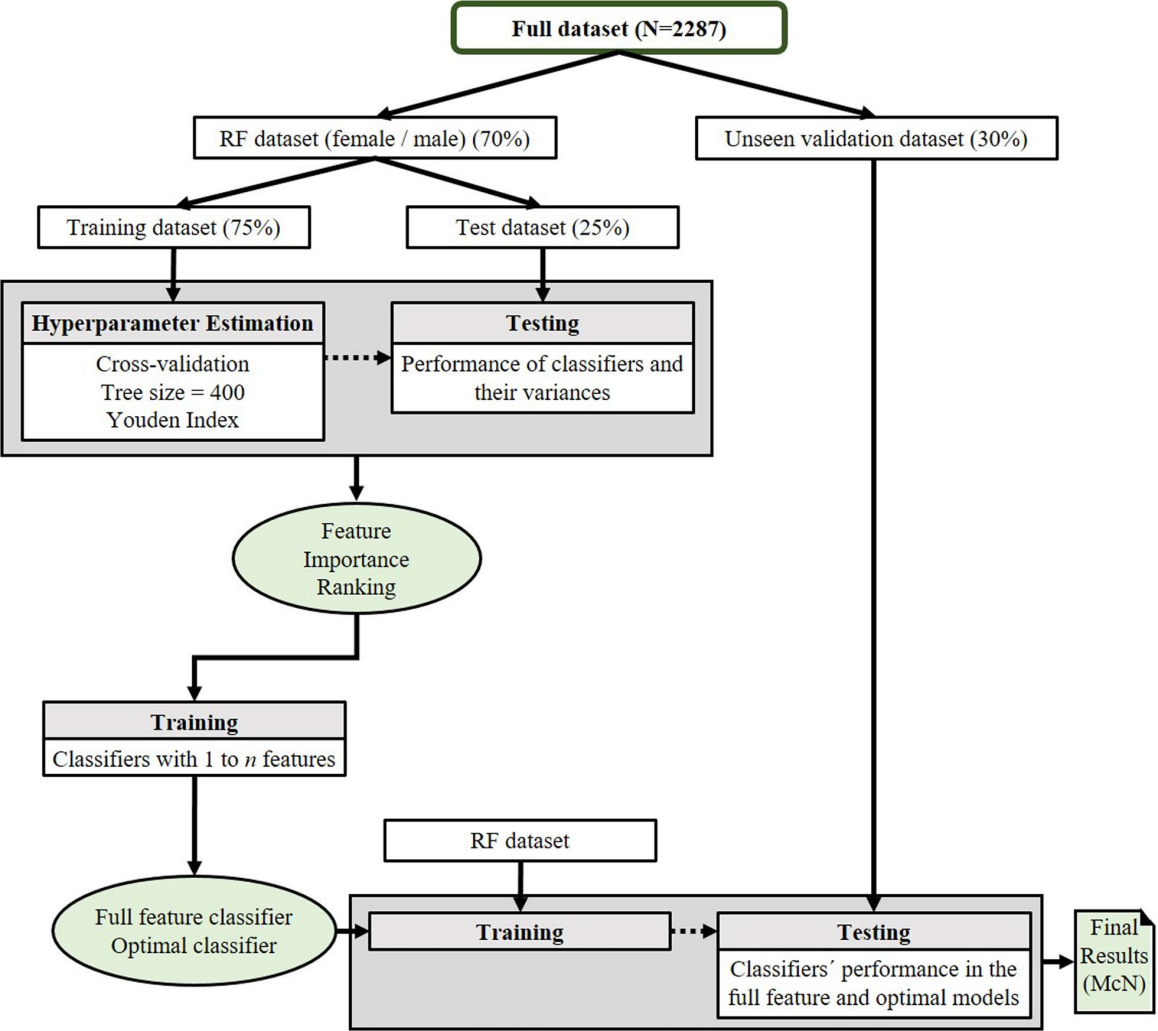
Flow diagram of the steps in the machine learning process

Potential biases due to site effects were tested by including site as a predictive feature in the RF. As it was of no importance, the final RF included only ADOS items. RFs are ensemble classifiers based on several decision trees aggregated by majority voting. Each decision tree yields a class prediction considering a random subset of features, and a majority vote of all the trees (“the forest”) forms the final classification [27]. Figure 1 summarizes the steps during training, testing, and validating the random forest.

We implemented guards against overfitting by splitting the total sample into a 70% RF (training and test) set and a 30% hold out set for validation of the final models. The RF set was again split into a 75% training set for model building and hyperparameter estimation and a 25% test set for model evaluation. The procedure consists of four consecutive steps, uses the R package *randomForest* [28] and is described in detail in [29]. The first step was a feature selection that gave us a hierarchy of all features regarding their importance for predicting class membership (i.e., ASD versus non-ASD). In a second step, we stepwise reduced the number of features that entered the training of the RF according to their importance rank. Reduced models were trained with 20-fold cross-validation using 95% of the data for training and 5% for testing. Subsequently, the reduced feature classifiers were evaluated on the previously held out and unseen validation data set, and the “optimal model” was determined by calculating a weighted ratio of accuracy and complexity (number of variables) for each model, with the choice of weights favoring simpler models in a 2:1 ratio (i.e., *w*1 * AUC + *w*2 * complexity where *w*1 = 0.35 and *w*2 = 0.65). Each model’s accuracy (ACC), sensitivity, and specificity are presented as indices of model quality. In the final step, the optimal model's predictive performance (accuracy) was statistically tested against the full features model using the McNemar test.

## Results

### Differences in symptom severity

In the *child and young adolescent sample with ASD*, no differences between females and males were observed concerning the social affect and RRB domains of the ADOS (see Table 2). However, the Calibrated Severity Score (CSS) was higher in males than in females (*T*(545) = 2.4, *p* = 0.016), with a small effect size (*d* = 0.33). In the ADI-R, we found differences in the Communication domain (*T*(370) = 2.5, *p* = 0.011) and the RRB domain (*T*(370) = 2.2, *p* = 0.026), with moderate effect sizes (*d* = 0.48 and 0.46).

In *older adolescents and adults with ASD*, differences were observed between females and males concerning the RRB domain of the ADOS (*T*(508) = 2.3, *p* = 0.023, *d* = 0.23) (see Table 2), but this difference did not emerge in the anamnestic interview (ADI-R). Males showed slightly more deficits in the Communication domain (*T*(233) = 2.4, *p* = 0.016), with only a small effect size (*d* = 0.34). In particular, adult females with ASD had significantly more comorbidities (e.g., depression, social phobia), whereas males with ASD had more attention deficit hyperactivity disorders. In the non-ASD samples, the females and males had a similar number of further diagnoses, but again, females were more likely to have anxiety disorders (F40-48 of ICD-10) and males were more likely to have attention deficit hyperactivity disorders.

### Diagnostic threshold of the ADOS

In the *child and young adolescent* sample, 80.8% of females and 87.6% of males diagnosed with ASD met the Autism Spectrum cut-off of the ADOS-2 classification algorithm. On the other hand, 15.7% of females and 18.8% of males without an ASD diagnosis also met the ADOS-2 Autism Spectrum cut-off. In the *older adolescent and adult sample*, 82.7% of females and 88.4% of males diagnosed with ASD met the cut-off, but so too did 12.7% of females and 28.4% males without an ASD diagnosis. The fourfold Table 3 presents the ADOS scores and diagnostic groups.

### Random forest (RF) analyses

**Fig. 2:**
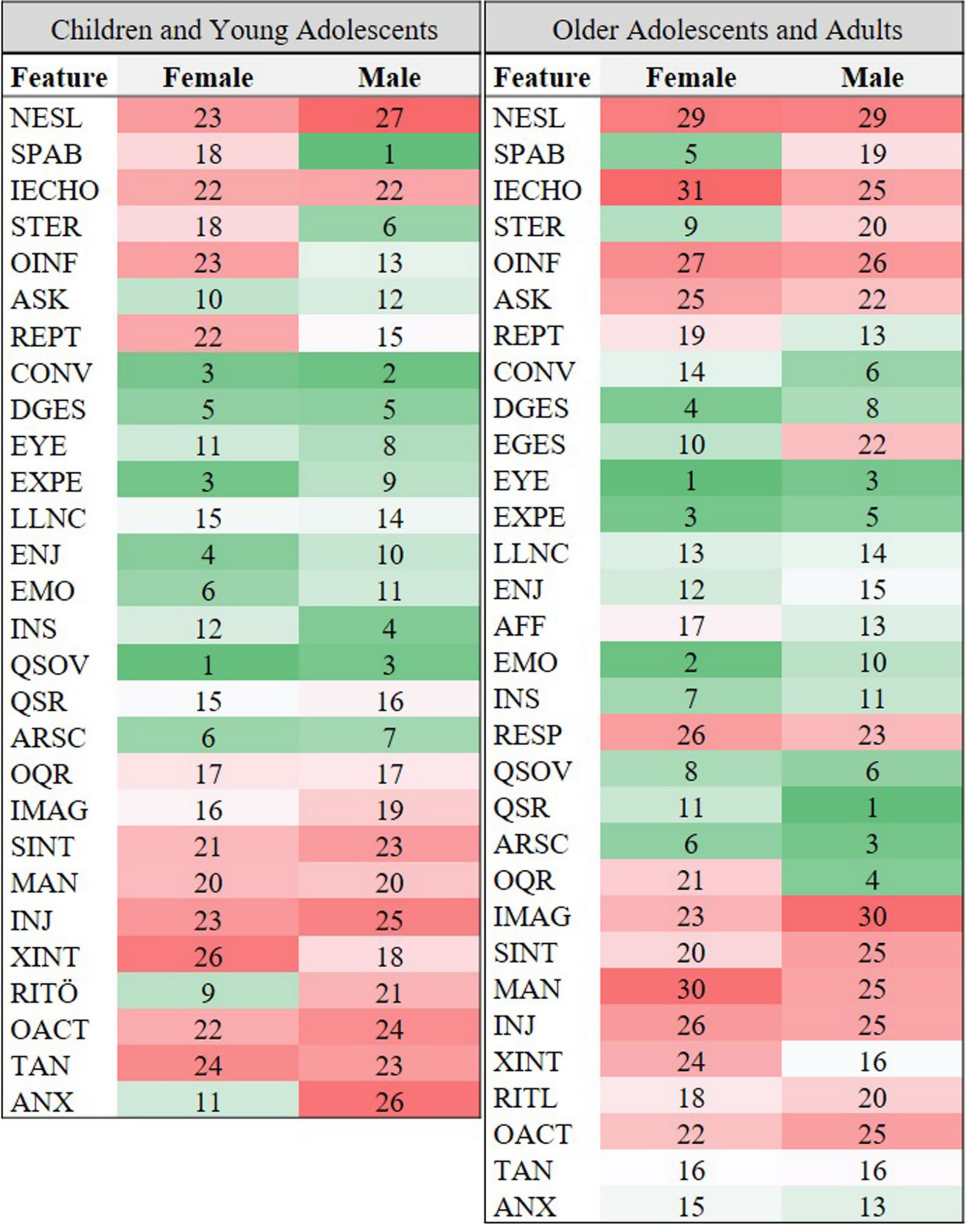
The average ranks of each feature in children and young adolescents and older adolescents and adults for visual comparison of the feature ranks between female and male individuals. *ANX* Anxiety, *ARSC* Amount of Reciprocal Social Communication, *ASK* Asks for Information, *CONV* Conversation, *DGES* Descriptive, Conventional, Instrumental, or Informational Gestures, *EMO* Empathy/Comments on Other’s Emotions, *ENJ* Shared Enjoyment in Interaction, *EXPE* Facial Expressions Directed to Examiner, *EYE* Unusual Eye Contact, *IECHO* Immediate Echolalia, *IMAG* Imagination/Creativity, *INJ* Self-Injurious Behavior, *INS* Insight, *LLNC* Language Production and Linked Nonverbal Communication, *MAN* Hand and Finger and Other Complex Mannerisms, *NESL* Overall Level of Non-Echoed Language, *OACT* Overactivity, *OINF* Offers Information, *OQR* Overall Quality of Rapport, *QSOV* Quality of Social Overtures, *QSR* Quality of Social Response, *REPT* Reporting of Events, *RITL* Compulsions or Rituals, *SINT* Unusual Sensory Interest in Play Material/Person, *SPAB* Speech Abnormalities Associated with Autism, *STER* Stereotyped/Idiosyncratic Use of Words or Phrases, *TAN* Tantrums, Aggression, Negative or Disruptive Behavior, *XINT* Excessive Interest in or References to Unusual or Highly Specific Topics or Objects or Repetitive Behaviors

The endorsement of ADOS items and their importance for the diagnostic classification (ASD versus non-ASD) was explored through a random forest approach for females and males separately. The first step of the analysis focused on identifying the latent feature importance ranking. Figure 2a, b shows the average rank of each feature from the cross-validation procedure in a heat map—comparing the ranking of features between sexes in children and young adolescents (Fig. 2a) and older adolescents and adults (Fig. 2b).

Model performance indices from the RF models are listed in Table 4. The behaviors associated with the optimal feature subsets are presented in Table 5 in descending order of importance.

**Table 4:** The performance of the ADOS diagnostic cut-off (= ADOS), performance of the RF models on the test set (= test) and the previously unseen validation data set (= val) for nonverbal children and young adolescents as well as older adolescents and adults

Module	No. of features	AUC ADOS	Sens. ADOS	Spec. ADOS	AUC test	ACC test	Sens. test	Spec. test	J	p McNe	AUC val	ACC val	Sens. val	Spec. val
Children and young adolescents	Female													
	ADOS algorithm	0.83	0.81	0.84										
	All 28 features				0.91	0.94	1	0.88	0.40		0.86	0.72	0.63	0.81
	5 features (optimal model)				0.92	0.93	0.97	0.91	0.43	.60	0.83	0.84	0.81	0.87
	Male													
	ADOS algorithm	0.87	0.88	0.81										
	All 28 features				0.93	0.89	0.93	0.86	0.44		0.79	0.86	0.85	0.88
	7 features (optimal model)				0.92	0.88	0.91	0.86	0.40	.14	0.81	0.85	0.85	0.85
Older adolescents and adults	Female													
	ADOS algorithm	0.89	0.82	0.88										
	All 31 features				0.83	0.88	0.91	0.82	0.46		0.92	0.83	0.93	0.72
	5 features (optimal model)				0.88	0.88	0.92	0.85	0.42	.18	0.86	0.78	0.84	0.72
	Male													
	ADOS algorithm	0.85	0.85	0.72										
	all 31 features				0.82	0.82	0.83	0.81	0.55		0.87	0.79	0.80	0.77
	8 features (optimal model)				0.82	0.80	0.84	0.76	0.48	.43	0.82	0.76	0.81	0.71

*AUC* area under the curve, *ACC* accuracy, *Sens.* sensitivity; *Spec.* specificity, *J* Youden’s index, *McN* McNemar level of significance—each model tested against the full-feature sets of available features

**Table 5:** The ADOS codes of behaviors identified as the optimal feature subset in descending order of importance for males and females

Female	Male
*Children and young adolescents*	
(1) ***Quality of Social Overtures (QSOV)*** (2) **Facial Expressions Directed to Examiner (EXPE)** (3) ***Conversation (CONV)*** (4) **Shared Enjoyment in Interaction (ENJ)** (5) ***Descriptive, Conventional, Instrumental or Informational Gestures (DGES)***	(1) Speech Abnormalities Associated with Autism (SPAB) (2) ***Conversation (CONV)*** (3) ***Quality of Social Overtures (QSOV)*** (4) Insight into Typical Social Situations and Relationships (INS) (5) ***Descriptive, Conventional, Instrumental or Informational Gestures (DGES)*** (6) **Amount of Reciprocal Social Communication (ARSC)** (7) **Stereotyped/Idiosyncratic Use of Words or Phrases (STER)** (8) **Unusual Eye Contact (EYE)**
*Older adolescents and adults*	
(1) ***Unusual Eye Contact (EYE)*** (2) Comments on others’ emotions/empathy (EMO) (3) ***Facial Expressions Directed to Examiner (EXPE)*** (4) *Descriptive, Conventional, Instrumental or Informational Gestures (DGES)* (5) **Speech Abnormalities Associated With Autism (SPAB)**	(1) **Quality of Social Responses (QSR)** (2) **Amount of Reciprocal Social Communication (ARSC)** (3) ***Unusual Eye Contact (EYE)*** (4) **Overall Quality of Rapport (OQR)** (5) ***Facial Expressions Directed to Examiner (EXPE)*** (6) **Quality of Social Overtures (QSOV)** (7) **Conversation (CONV)** (8) *Descriptive, Conventional, Instrumental or Informational Gestures (DGES)*

Bold font indicates items are comprised in the diagnostic algorithms (ADOS-2). Overlap between the sexes is written in italics

*Children and Young Adolescents* By utilizing the importance hierarchy from the feature selection, RFs for one to 28 features were calculated and evaluated on the test data separately for the two sexes. In *females*, the model output from the test set, including all 28 variables, showed an AUC of 0.91 with 1.00 sensitivity and 0.88 specificity. For independent validation of the classifier, its performance on the validation set was computed and yielded an AUC of 0.86 with 0.63 sensitivity and 0.81 specificity (see also Table 4 for an overview). A model including five features yielded optimal results in the validation set, with an AUC of 0.83 with 0.81 sensitivity and 0.87 specificity. The optimal model comprised the following features: Quality of Social Overtures (QSOV), Facial Expressions Directed to Examiner (EXPE), Conversation (CONV), Shared Enjoyment in Interaction (ENJ), Descriptive, Conventional, Instrumental, or Informational Gestures (DGES). A comparison of the models’ performance via McNemar’s test for differences in classification error rates showed no advantage of the full-feature model (28 features) over the weighted optimal model with five features (*χ*^2^ = 0.266, *p* = 0.60).

In *males*, the model output from the test set, including all 28 variables, showed an AUC of 0.93 with 0.93 sensitivity and 0.86 specificity. For independent validation of the classifier, its performance on the validation set was computed, and yielded an AUC of 0.79 with 0.85 sensitivity and 0.88 specificity. A model including eight features yielded optimal results in the validation set, with an AUC of 0.81 and 0.85 sensitivity and 0.85 specificity. The following eight features were included in the optimal model: Speech Abnormalities Associated With Autism (SPAB), Conversation (CONV), Quality of Social Overtures (QSOV), Insight Into Typical Social Situations and Relationships (INS), Descriptive, Conventional, Instrumental, or Informational Gestures (DGES), Amount of Reciprocal Social Communication (ARSC), Stereotyped/Idiosyncratic Use of Words or Phrases (STER), Unusual Eye Contact (EYE). A comparison of the models’ performance via McNemar’s test for differences in classification error rates showed no advantage of the full-feature model (28 features) over the weighted optimal model with eight features (*χ*^2^ = 0.209, *p* = 0.14).

*Older Adolescents and Adults* By utilizing the importance hierarchy from the feature selection, RFs for one to 31 features were calculated and evaluated on the test data separately for the two sexes. In *females*, the model output from the test set, including all 31 variables, showed an AUC of 0.83, with 0.91 sensitivity and 0.82 specificity. For independent validation of the classifier, its performance on the validation set was computed, and yielded an AUC of 0.92, with 0.93 sensitivity and 0.72 specificity (see also Table 4 for an overview). A model including five features yielded optimal results in the validation set with an AUC of 0.86, with 0.84 sensitivity and 0.72 specificity. The optimal model comprised the following features: Unusual Eye Contact (EYE), Comments on Others’ Emotions/Empathy (EMO), Facial Expressions Directed to Examiner (EXPE), Descriptive, Conventional, Instrumental, or Informational Gestures (DGES), Speech Abnormalities Associated With Autism (SPAB). A comparison of the models’ performance via McNemar’s test for differences in classification error rates showed no advantage of the full-feature model (31 features) over the weighted optimal model with five features (*χ*^2^ = 1.76, *p* = 0.18).

In *males*, the model output from the test set, including all 31 variables, showed an AUC of 0.82, with 0.83 sensitivity and 0.81 specificity. For independent validation of the classifier, its performance on the validation set was computed, and yielded an AUC of 0.87, with 0.80 sensitivity and 0.77 specificity. A model including eight features yielded optimal results in the validation set, with an AUC of 0.87 and 0.80 sensitivity and 0.77 specificity. The optimal model comprised the following features: Quality of Social Responses (QSR), Amount of Reciprocal Social Communication (ARSC), Unusual Eye Contact (EYE), Overall Quality of Rapport (OQR), Facial Expressions Directed to Examiner (EXPE), Quality of Social Overtures (QSOV), Conversation (CONV), Descriptive, Conventional, Instrumental, or Informational Gestures (DGES). A comparison of the models’ performance via McNemar’s test for differences in classification error rates showed no advantage of the full-feature model (31 features) over the weighted optimal model with eight features (*χ*^2^ = 0.622, *p* = 0.43).

## Discussion

The present study aimed to explore potential differences in how female and male individuals meet the diagnostic criteria for ASD assessed by the ADOS. We aimed to identify a potential female phenotype from behavioral observations in a well-characterized clinical population of children, adolescents, and adults. Using a random forest approach, we compared subsets of diagnostic features of the ADOS that were most indicative of an ASD diagnosis between females and males.

### Overall results

The results revealed similar classifier performances in the female and male samples, but relying on slightly different features for classification. Concentrating on a few core behavioral aspects for female and male samples led to classification performances that were equally as good as those based on information from the complete examination. For an optimal performance, the classifiers needed fewer features in the female sample than in the male sample in both age groups. It has been argued that since the defining diagnostic criteria are historically based on the male phenotype and the diagnostic thresholds are similar, a female phenotype may be missed if it presents differently, even if these females present with a substantial clinical burden [7]. However, the current study demonstrates that although slightly different features were most discriminative, classification in females was just as good as in males.

### Differences in symptom severity

In the current study, females were older at the time of the diagnostic appointment—an effect that was pronounced in the *older adolescent and adult sample*. In the *young adolescent and adult group*, males with ASD scored higher in the RRB domain of the behavior observation than females with ASD, but the effect size was small. We observed no differences in social affect between the sexes in the ASD samples of either age group, but males scored higher on overall symptom severity. These findings are in line with a meta-analysis that reported few differences in communication and social behavior between males and females and only in the RRB domain did girls show fewer symptoms than boys [18].

The present findings indicate, however, that as ASD symptoms present differently across development, the developmental aspect might be important with respect to sex differences: In the *older adolescent and adult sample*, we found fewer symptoms of RRB and lower overall ASD severity (ADOS CSS total) in females than in males. From the parental perspective (anamnestic data from the ADI-R), females showed fewer symptoms in the communication domain. In the *child and young adolescent sample*, more parent-reported RRB were observed in males compared to females, with moderate effect sizes. Classification accuracy of the RF models was similar to the diagnostic accuracy of the ADOS-2 algorithm in females as well as males. Interestingly, we found more females than males who were diagnosed with ASD while scoring below the ADOS autism spectrum diagnostic cut-off (18.6% females vs. 13.5% males, i.e., false negative ADOS classifications). This suggests that information from outside the standardized behavioral observation may be of greater importance for the diagnostic decision in females than in males, giving rise to the question of which particular additional information clinicians rely on in order to classify a female as autistic. On the other hand, more males than females did not receive an ASD diagnosis despite exceeding the ADOS diagnostic threshold (6.4% females vs. 14.2% males, i.e., false positive ADOS classifications). This suggests that autistic traits in males may be present during the behavioral observation but are attributed to other underlying conditions or symptoms of a differential diagnosis. However, our female sample had more comorbid diagnoses (e.g., depression, social phobia), and particularly in females with ASD, there is evidence that the presence of depression and anxiety is associated with enhanced ASD symptoms [30–35]. The considerable symptom overlap of ASD with depressive and anxiety disorders entails the risk of false-positive evaluations in females. Although the ADOS-2 shows high sensitivity (0.91; [23], p. 243) for detecting autism versus non-spectrum cases, emerging research shows that it may be less accurate in detecting ASD in individuals with complex psychiatric presentations [36]. Moreover, the observation in the current sample that the prevalence of ASD diagnoses increases with age (45.9% of all *adolescent/ adult* females, but only 33.8% of the younger sample, received an ASD diagnosis) underlines the need to carefully consider differential, potentially overlapping diagnoses during the diagnostic process.

### Differences in diagnostic features of the ADOS

To the best of our knowledge, this is the first study to explore sex differences in an ASD and a non-ASD sample with the aim of identifying those symptoms that are most important for the classification and subsequently comparing these discriminative features between females and males. The most discriminative features all stem from the social communication domain of the ADOS, whereas only speech items (Speech Abnormalities Associated with Autism, and Stereotyped and Idiosyncratic Use of Words or Phrases) of the RBB domain are included in the optimal feature models. This may be due to the rather short time span of the ADOS (45–60 min duration of administration), which limits the time for observations of repetitive behaviors and/or the overall more verbal character of the ADOS modules 3 and 4. Furthermore, although males showed more RBBs than females, the pattern in male and female non-ASD cases seemed similar thus not providing the RF classifier information relevant for the distinction of ASD and non-ASD cases within each group. The effect may also be attributed to basic sex differences in the occurrence of RRB in the diagnostic situation elicited in boys by a male-biased toy selection. As has been pointed out, the restricted and repetitive interests among females may be more “random” and more difficult to categorize and thus to “identify as atypical” [15, p. 1391].

Our optimal models include mainly ADOS items mapping onto “Basic Social Communication Skills.” According to Bishop and colleagues [37], social communication deficits captured by the ADOS can be divided into “Basic Social Communication Skills” (including Gestures, Eye Contact, Facial Expressions, and Shared Enjoyment) and “Interaction Quality” (including Conversation, Amount of Reciprocal Social Communication, and other Quality items). These ‘basic’ impairments seem to be specific for ASD regardless of sex, age, and intelligence [37, 38]. In our models, these basic impairments appear, overall, to be sufficient in order to discriminate females with ASD from those with other mental disorders when flanked by the two additional items of “Interaction Quality,” with good specificity and sensitivity. Moreover, in contrast to the findings for males, they are not correlated with age and IQ (see Additional file 1: Table S3). Some previous studies found that females with ASD exhibit less severe impairments in social communication behaviors [39, 40], although we and others [7, 14] cannot confirm this for the behavior observation. Nevertheless, these items do seem to be essential for the differentiation of ASD from other mental disorders, particularly in females.

In the *child and young adolescent sample*, we found similarities between females and males concerning the following items: Quality of Social Overtures, Conversation, and Gestures. Differences were especially evident in the communication domain. Speech abnormalities were also relevant for the differentiation from other mental disorders. Such speech abnormalities are important for females: For the female group, all items are algorithm items, whereas for the male group, six items are part of the algorithm and two additional items are needed (Speech Abnormalities and Insight) for the model to reach optimal classification performance. *In the older adolescent and adult sample,* similarities were only found concerning the basic skills Eye Contact, Facial Expressions, and Gestures. However, for the differentiation from other mental disorders in males, many aspects of the Quality of Interaction are additionally needed; in females, only Empathy and Speech Abnormalities are relevant.

In the male, the most discriminative ADOS items all stem from the classification algorithm plus the item Descriptive, Conventional, Instrumental or Informational Gestures (DGES). In the female sample, though a smaller number of features seem to suffice for an optimal classification, only 3 out of 5 items stem from the ADOS classification algorithm. Particularly, the item Comments on Others’ Emotions/Empathy that is linked to cognitive empathy, a construct often impaired in ASD [41], was of prime importance in the optimal model.

Overall, the optimal models of our RF approach yielded slightly different distinctive features for females and males but did not outperform the ADOS-2 classification algorithm (grouping the autism spectrum and autism cases together). These results do not suggest an adaptation of the ADOS-2 classification algorithm for a female phenotype.

Future aim of the present work is to break down these most discriminative subsets of diagnostic items into their underlying mechanisms or processes and translate them into research on biomarkers in order to identify the behaviorally observed differences between females and males on a molecular level. This needs to be the next step on the way to the identification of a female phenotype as both measures—ADOS and ADI-R—cannot simply be abbreviated, as, e.g., ADOS codes are attained throughout the observation session and are not strictly tied to single subtasks [24] and thus items cannot be observed independently and the impact of each item for the diagnostic decision is difficult to extract.

## Strengths and limitations

The observation of differences between the sexes not only regarding the most discriminating diagnostic features but also across the age groups leads to the assumption that gender associated symptom presentation changes during development. Future studies therefore need to evaluate sex differences in younger age groups and ideally in longitudinal studies in children at risk, who are eventually diagnosed with ASD or other developmental or clinical conditions. Only longitudinal data can clarify “age differences in how ASD manifests in boys vs. girls, from other phenotypic differences” [14], p. 102).

A particular strength of the present study lies in the composition of the sample. Previous research on sex/gender effects only included individuals with a confirmed ASD diagnosis, and may therefore have missed females with different symptom profiles (the “female phenotype”). By contrast, the present study investigated a broader clinical sample that also included individuals with suspicion of ASD. This had the advantage that we were able to evaluate sensitivity and specificity, and did not merely treat scores as indices of symptom severity, as was the case in previous studies [7, 14]. Thus, it was possible to evaluate the utility of standard instruments also among individuals with autistic traits but with other mental disorders. In turn, this enabled us to identify symptom profiles in females that led to a diagnostic decision and to compare them to symptom profiles in males. Furthermore, diagnoses in the present study were best estimate clinical diagnoses (BEC) and did not solely rely on the diagnostic thresholds of the “gold standard” instruments (ADOS and ADI-R). The sample thus included individuals (female and male alike) who did not meet the ADOS/ADI-R cut-offs but were nevertheless diagnosed with ASD, or conversely, individuals who were not diagnosed with ASD despite their scores exceeding the diagnostic threshold.

As was the case in previous studies, our sample of females with ASD was smaller than the male sample. Therefore, females may still be underrepresented, limiting the statistical power to detect small to moderate effects. A further limitation concerns our study design: Although ASD diagnoses in the current study were BEC diagnoses and did not rely solely on ADOS scores, these scores were nevertheless employed as part of the diagnostic assessment, leading to a certain degree of circularity. This is also associated to the limitation that behaviors captured by the ADOS might already be male-biased because the development and validation of the instrument were undertaken with predominantly male cases. We tackled this by relying on BEC diagnoses that included multiple sources of information a mere classification based on ADOS (and ADI-R) cut-off scores. We thus have individuals in the sample that scored beyond cut-off but were nevertheless diagnosed with ASD and individuals that exceeded the cut-off but were not diagnosed with ASD. In order to approach this limitation, future studies need to extend the methodological approach to data-driven analyses. Previous studies have pursued subgroups within the autism spectrum and were able to identify subgroups based on social interaction and communication, intelligence, and morphological abnormalities. However, behavioral subgroups have not yet been replicated [42] and sex or gender has not yet been taken into account.

## Conclusion

Altogether, we found similarities and some differences between females and males with ASD. The reduced feature models in females relied on considerably fewer features from the ADOS than those in males, while still yielding similar classification performances. Although we identified some phenotypic differences between females and males with ASD, the existing diagnostic ADOS algorithm seems to be sufficient to capture the core diagnostic criteria in females and males. These results lead to the conclusion that the available standardized behavior observation (ADOS) should remain a substantial part of the diagnostic procedure and that clinicians need to be aware of potential differential diagnoses, particularly in females.

## Supplementary Information

**Additional file 1**. **Table S1**. Psychopathological characterization of children and young adolescents: All ICD-10 diagnoses are listed, including comorbidities, separated for sex. **Table S2**. Psychopathological characterization of Older Adolescents/Adults—all ICD-10 diagnoses are listed, including comorbidities, separated for sex. **Table S3**. Pearson correlations between optimal feature set, age and IQ.

## Data Availability

The data are not publicly available due to medical confidentiality but are available from the first author on request pending the approval of the coauthors.

## Abbreviations

ACC: Accuracy
ADHD: Attention-deficit hyperactivity disorder
ADI-R: Autism diagnostic interview-revised
ADI-R A: Social interaction domain
ADI-R B: Communication domain
ADI-R C: Restricted repetitive behaviors domain
ADOS: Autism Diagnostic Observation Schedule
ARSC: Amount of Reciprocal Social Communication
ASD: Autism spectrum disorder
AUC: Area under the curve
BEC: Best estimate clinical diagnosis
CONV: Conversation
CSS: Comparison score
DGES: Descriptive, Conventional, Instrumental, or Informational Gestures
EMO: Comments on Others’ Emotions/Empathy
ENJ: Shared Enjoyment in Interaction
ES: Effect size (Cohen’s d)
EXPE: Facial Expressions Directed to Examiner
EYE: Unusual Eye Contact
INS: Insight Into Typical Social Situations and Relationships
IQ: Intelligence Quotient
J: Youden’s Index
McN: McNemar level of significance
OQR: Overall Quality of Rapport
RF: Random forest
RRB: Restricted and Repetitive Behaviors
QSOV: Quality of Social Overtures
QSR: Quality of Social Responses
SA: Social affect
Sens.: Sensitivity
SPAB: Speech Abnormalities Associated with Autism
Spec: Specificity
STER: Stereotyped/Idiosyncratic Use of Words or Phrases
