# Supplementary material for: Phenotypic differences between female and male individuals with suspicion of autism spectrum disorder
Source: Mol Autism. 2022 Mar 7;13:11. doi: 10.1186/s13229-022-00491-9 (PMC8900413; doi:10.1186/s13229-022-00491-9)
Supplement: Supplementary file 1 — Additional file 1. Table S1. Psychopathological characterization of children and young adolescents: All ICD-10 diagnoses are listed, including comorbidities, separated for sex. Table S2. Psychopathological characterization of Older Adolescents/Adults—all ICD-10 diagnoses are listed, including comorbidities, separated for sex. Table S3. Pearson correlations between optimal feature set, age and IQ. [file 13229_2022_491_MOESM1_ESM.pdf]

Table S1. Psychopathological characterization of **children and young adolescents:** All ICD-10 diagnoses are listed, including comorbidities, separated for sex. The table presents absolute numbers with percent in brackets per group.

**Psychopathological characterization**		**ASD N=547**	**Non-ASD N= 805**
	male	495	703
	female	52	102
F20-F29	Schizophrenia, schizotypal and delusional disorders	4 (0.7%)	4 (0.5%)
	male	4 (0.8%)	4 (0.5%)
	Female	0 (0%)	0 (0%)
F30-F39	Affective disorders (most common: Recurrent depressive disorder)	0 (0%)	3 (0.4%)
	male	0(0%)	3 (0.4%)
	female	0 (0%)	0 (0%)
F40-F48	Neurotic, stress-related and somatoform disorder (most common: Obsessive-compulsive disorder)	10 (1.8%)	24 (3.0%)
	male	9 (1.8%)	21 (3.0%)
	female	1 (1.9%)	3 (2.9%)
F50-F59	Behavioral syndromes associated with physiological disturbances and physical factors	0 (0%)	5 (0.6%)
	male	0 (0%)	4 (0.6%)
	female	0 (0%)	1 (1%)
F60-F69	Disorders of adult personality and behavior	1 (0.2%)	5 (0.6%)
	male	1 (0.2%)	4 (0.6%)
	female	0 (0%)	1 (1%)
F70-F79	Mental retardation with impairment of behavior	25 (5.4%)	45 (6.7%)
	male	21(4.2%)	**33 (4.7%)**
	female	4 (7.7%)	**12 (11.8%)****
F80–F89 *except* F84	Disorders of psychological development other than pervasive developmental disorders	132 (24.2%)	197 (24.5%)
	male	118 (23.8%)	174 (24.8%)
	female	14 (26.9%)	23 (22.6%)
F90-F98	Behavioral and emotional disorders with onset usually occurring in childhood and adolescence (most common: Hyperkinetic disorders)	157 (28.6%)	550 (68.2%)
	male	144 (29.1%)	488 (69.3%)
	female	13 (25%)	62 (60.9%)
Other	Most common: mental and behavioral disorders due to psychoactive substance use	1 (0.2%)	5 (0.6%)
	male	1 (0.2%)	4 (0.6%)
	female	0 (0%)	1 (1%)
Cases with *further* (axis one or two) ICD-10 diagnoses		277 (50.6%)	630 (78.3%)
	male	246 (49.7%)	553 (78.7%)
	female	31 (59,6%)	77 (75.5%)
Cases with no (axis one) ICD-10 diagnoses			175 (21,7%)
	male		150 (21.3%)
	female		25 (24.5%)

ASD=autism spectrum disorder; **= significant difference between the sexes according to Chi-square test ($p<.05$)

Table S2. Psychopathological characterization of **Older Adolescents/Adults**– all ICD-10 diagnoses are listed, including comorbidities, separated for sex. The table presents absolute numbers with percent in brackets per group.

**Psychopathological characterization**		**ASD N=510**	**Non-ASD N= 425**
	male	371	307
	female	139	118
F20-F29	Schizophrenia, schizotypal and delusional disorders	4 (0.8%)	10 (2.4%)
	male	2 (0.5%)	8 (2.6%)
	Female	2 (1.4%)	2 (1.7%)
F30-F39	Affective disorders (most common: Recurrent depressive disorder)	126 (24.7%)	89 (21%)
	male	**77 (20.8%)****	64 (20.8%)
	female	**49 (35.3%)****	25 (21.2%)
F40-F48	Neurotic, stress-related and somatoform disorder (most common: social phobia)	79 (15.5%)	86 (20.2%)
	male	**41 (11.0%)****	**56 (17.9%)****
	female	**38 (27.4%)****	**31 (26.2%)****
F50-F59	Behavioral syndromes associated with physiological disturbances and physical factors	2 (0.4%)	1 (0.2%)
	male	1 (0.3%)	0
	female	1 (0.7%)	1 (0.8%)
F60-F69	Disorders of adult personality and behavior (most common: Personality disorder)	21 (4.1%)	87 (20.5%)
	male	12 (3.2%)	63 (20.5%)
	female	9 (6.5%)	24 (20.3%)
F80–F89 *except* F84	Disorders of psychological development other than pervasive developmental disorders	31 (6.1%)	33 (7.8%)
	male	25 (6.7%)	31 (10.1%)
	female	6 (4.3%)	2 (1.7%)
F90-F98	Behavioral and emotional disorders with onset usually occurring in childhood and adolescence (most common: Hyperkinetic disorders)	55 (10.8%)	86 (20.2%)
	male	**50 (13.5%)****	**72 (23.4%)****
	female	**5 (3.6%)****	**14 (11.9%)****
Other	Most common: mental and behavioral disorders due to psychoactive substance use	15 (2.9%)	18 (4.3%)
	male	10 (2.7%)	14 (4.5%)
	female	5 (3.6%)	4 (3.3%)
Cases with *further* (axis one or two) ICD-10 diagnoses		259 (51%)	307 (72.2%)
	male	**177 (47.7%)**	228 (74.3%)
	female	**82 (59.0%)****	79 (66.9%)
Cases with no (axis one) ICD-10 diagnoses			151(35.2%
	male		106(34.5%)
	female		45(38.1%)

ASD=autism spectrum disorder; ** significant difference between the sexes according to Chi-square test (p<.05)

Table S3. Pearson correlations between optimal feature set, age and IQ

	Female	male
Children and Young Adolescents		
age	.134	.036
IQ	-.032	-.096**
Older Adolescents and Adults		
age	.010	-.105**
IQ	.107	-.179**

IQ = intelligence quotient
